# An experimental pleural drainage device in hypertensive pneumothorax

**DOI:** 10.1590/ACB360708

**Published:** 2021-08-23

**Authors:** Bruno Filipe Viotto Petta, Renato Fernando Cazanti, Carlos Edmundo Rodrigues Fontes

**Affiliations:** 1 Fellow Master degree. Postgraduate Program in Management, Technology and Innovation in Emergency and Urgency – Department of Medicine - Universidade Estadual de Maringá – Maringa (PR), Brazil; 2 Fellow Master degree. Postgraduate Program in Management, Technology and Innovation in Emergency and Urgency - Department of Medicine - Universidade Estadual de Maringá – Maringa (PR), Brazil.; 3 Post-Doctorate. University of Paris XI Paul Bousse Hospital and Department of Medicine - Universidade Estadual de Maringá – Maringa (PR), Brazil.

**Keywords:** Pneumothorax, Trauma, Pulmonary Surgical Procedures, Printing, Three-Dimensional

## Abstract

**Purpose:**

To develop a specific device for pleural drainage in hypertensive pneumothorax.

**Methods:**

The prototype was modeled from the free version of a 3D modeling application, printed on a 3D printer using ABS^®^ plastic material, and tested in a pleural drainage simulator.

**Results:**

Pleural drainage in the simulator using the prototype was feasible and reproducible.

**Conclusions:**

While the prototype is functional in the simulator, it requires improvement and refinement for use in humans.

## Introduction

Data from the World Health Organization in 2010 estimated that trauma was responsible for the deaths of nine people per minute, 5.8 million deaths per year, and 12% of the cost of diseases throughout the world. Chest trauma accounted for 20% of all traumas in general and was responsible for 20-25% of all trauma-related deaths^1^.

Life-threatening thoracic injuries can be treated with airway control or chest decompression with a needle, followed by digital or tubular drainage^2^^,^ 3. Hypertensive pneumothorax is a lesion caused by the combination of a traumatic air fistula and a unidirectional valve mechanism, resulting from penetrating blunt or iatrogenic trauma. There is no air leak; the air accumulates in the pleural space, compressing the ipsilateral lung with a deviation of the contralateral mediastinum, decreased venous return and cardiac output, and consequent obstructive cardiac shock^4^. Once diagnosed, thoracic decompression is mandatory to prevent progression to death^3^^,^ 4.

The ninth edition of the Advanced Trauma Life Support (ATLS) recommended the insertion of a 5-cm angiocatheter at the point located between the second intercostal space and the hemiclavicular line. However, the tenth and latest edition of the ATLS recommended insertion between the fourth or fifth intercostal space and between the anterior axillary line and the middle axillary line. This change resulted from studies that demonstrated failure rates between 4-65% in needle drainage, either by incompatibility between the length of the angiocatheter and the thickness of the chest wall^5^, by mechanical obstruction of the lumen, or by inaccurate anatomical location by the physician^6^.

No further device has been specifically developed for chest decompression since the introduction of the angiocatheter. Furthermore, the latter is not considered a definitive procedure since it requires thoracostomy with tubular pleural drainage in a water seal. The objective of this study was, therefore, to propose a specific device for pleural drainage in hypertensive pneumothorax.

## Methods

Through the free version of a tridimensional (3D) modeling application (Shapr3D®), a digital model of the device’s parts was developed, and they were printed on a 3D printer using acrylonitrile butadiene styrene (ABS) plastic. The device consists of three parts: the main body, the release body, and the piercing rod. The main body is cylindrical, 185-mm long and 40 mm in diameter and has grooves to consolidate grip and prevent the device from slipping while handling. The released body, in turn, is composed of a 35-mm diameter and 15-mm long cylindrical piece, connected to a 20-mm long by 10-mm wide and 5-mm thick feed button. Finally, the perforating-cutting rod is 125-mm long and 5 mm in diameter, and the 5-mm distal portion has triangular shape and 2-mm thickness.

The printed model was tested in a pleural drainage simulator with a porcine rib, using an 8.5-Fr tracheal cannula as a pleural drain. The technique for pleural drainage was based on the ATLS precepts. After identifying the intercostal space and performing local anesthesia, the device containing the 8.5-Fr tracheal cannula over the perforating-cutting rod was pressed against the intercostal space up to the physical limitations of the device, allowing the skin and soft tissues to be cut up to 65 mm of the rod. Then, the advancement button was actuated, allowing the progression of another 20 mm of the rod and the implantation of the tracheal cannula in the pleural space. The device was pulled and removed, and the balloon of the cannula was inflated with 20 mL of air and fixed to the skin with 2-0 nylon.

## Results

**Figure 1:**
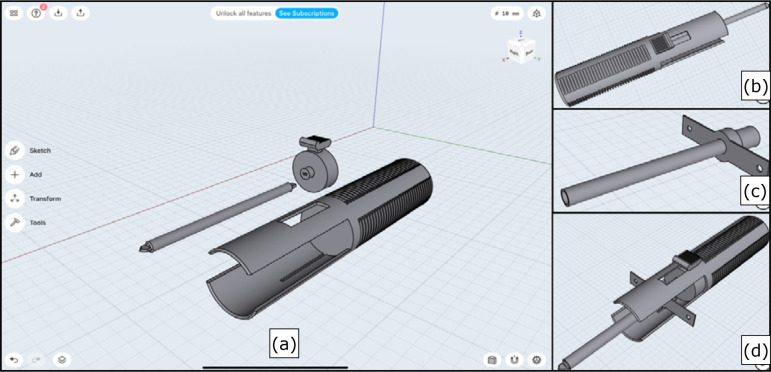
(**a**) The three parts of the device developed using the 3D modeling application (Shapr3D^®^); (**b**) The 3D model with the three parts assembled; (**c**) 3D modeling of the tracheal cannula; (**d**) 3D model of the device completely assembled with the tracheal cannula.

**Figure 2:**
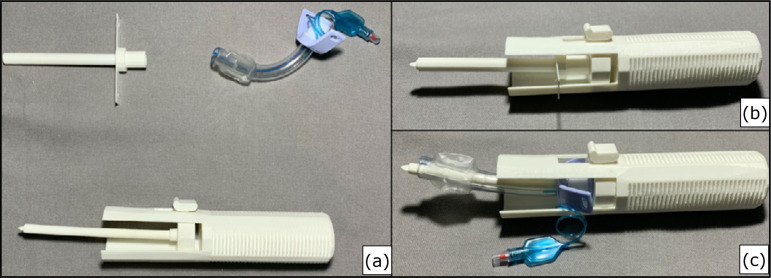
(**a**) Printed device, printed tracheal cannula, and real tracheal cannula no. 8.5-Fr; (**b**) device completely assembled with the printed tracheal cannula; (**c**) device wholly assembled with the real tracheal cannula no. 8.5-Fr.

**Figure 3:**
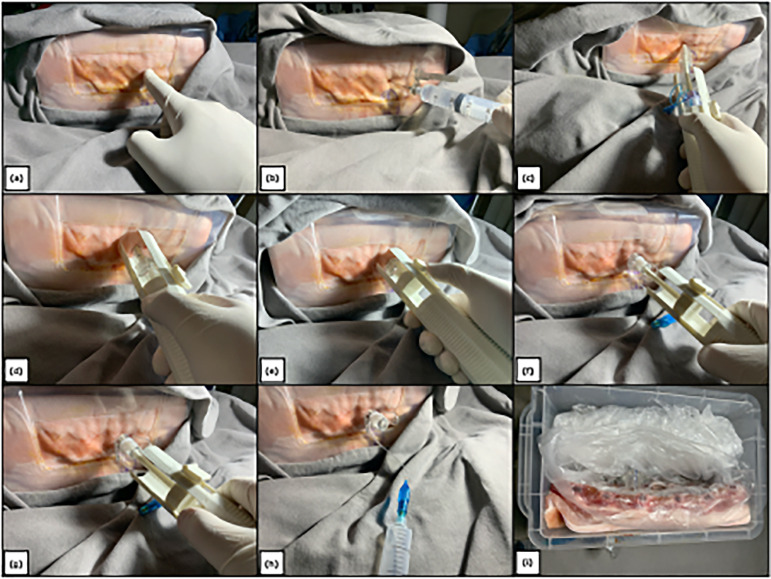
Diagram of the pleural drainage procedure in hypertensive pneumothorax using the proposed device. (**a**) Identification of the intercostal space; (**b**) local anesthetic block; (**c**) puncture of the intercostal space using the device; (**d**) insertion of the device up to the physical limit; (**e**) activation of the advancement button; (**f and g**) release of the cannula; (**h**) inflation of the cannula balloon; (**i**) verification and confirmation of pleural drainage after the procedure on the simulator.

The results are presented in Figs 1-3:

3D: tridimensional.

## Discussion

Needle puncture is the first action taken after the diagnosis of hypertensive pneumothorax^7^. It is generally a procedure performed in a pre-hospital setting or in life-threatening situations in the emergency room or intensive care unit. It aims to transform the potentially fatal hypertensive pneumothorax into a simple pneumothorax that will need to be drained posteriorly^8^.

As it is performed in a stressful environment and often under unfavorable conditions, needle decompression is ineffective in more than 50% of cases, either because of the technique, or on account of the instrument used^8^^-^ 11. After needle decompression, simple pneumothorax requires definitive pleural drainage. Often, either due to a delay in hospital transport or ineffective decompression, the patient develops a new hypertensive pneumothorax.

Based on these problems, a pleural drainage device that is more effective than needle decompression was proposed and developed. It is a definitive treatment which does not require a second procedure.

Some features characterize the prototype. The length of the device and its release mechanism allow sufficient length of the intrapleural drain and overcome the chest wall thickness, even in patients with larger walls or obese patients. The main body of the device has a mechanical limiter that prevents the indiscriminate introduction of the perforating-cutting rod, thus preventing iatrogenic intra-parenchymal or vascular injuries. It allows the use of an 8.5-Fr tracheal cannula as a pleural tube. The lumen of the cannula has a diameter similar to pleural tubes ranging from 20-28-Fr, making possible the drainage of thick secretions such as blood and pus^12^. The length of the cannula prevents the kinking of the tube. The inflated balloon enables a quick fixation and sealing of periostomy air leakage. Finally, the cannula can be attached to a water seal system or to a Heimlich valve.

The prototype worked when tested in a pleural drainage simulator using porcine ribs. However, it may well benefit from adaptations, modifications, and improvements. A partnership with the medical-hospital supplies industry will allow improvement and diversification in the use of this device in its applications regarding pneumothorax, cricothyroidotomies, and tracheostomies in humans. Due to budget limitations, it was not possible to manufacture the perforating-cutting rod in surgical stainless steel. This is a limitation of our prototype.

## Conclusion

The device proved functional in the simulator. It has potential to be used as a definitive treatment for hypertensive pneumothorax, but it requires investment and enhancement.
